# Deletion of ameloblastin exon 6 is associated with amelogenesis imperfecta

**DOI:** 10.1093/hmg/ddu247

**Published:** 2014-05-23

**Authors:** James A. Poulter, Gina Murillo, Steven J. Brookes, Claire E. L. Smith, David A. Parry, Sandra Silva, Jennifer Kirkham, Chris F. Inglehearn, Alan J. Mighell

**Affiliations:** 1 Leeds Institute of Biomedical and Clinical Sciences, St James's University Hospital, University of Leeds, Leeds LS9 7TF, UK; 2 School of Dentistry and; 3 Biology, Molecular Cellular Centre (CBCM), University of Costa Rica, San Pedro, Costa Rica; 4 School of Dentistry, University of Leeds, Leeds LS2 9LU, UK

## Abstract

Amelogenesis imperfecta (AI) describes a heterogeneous group of inherited dental enamel defects reflecting failure of normal amelogenesis. Ameloblastin (AMBN) is the second most abundant enamel matrix protein expressed during amelogenesis. The pivotal role of AMBN in amelogenesis has been confirmed experimentally using mouse models. However, no *AMBN* mutations have been associated with human AI. Using autozygosity mapping and exome sequencing, we identified genomic deletion of *AMBN* exon 6 in a second cousin consanguineous family with three of the six children having hypoplastic AI. The genomic deletion corresponds to an in-frame deletion of 79 amino acids, shortening the protein from 447 to 368 residues. Exfoliated primary teeth (unmatched to genotype) were available from family members. The most severely affected had thin, aprismatic enamel (similar to that reported in mice homozygous for *Ambn* lacking exons 5 and 6). Other teeth exhibited thicker but largely aprismatic enamel. One tooth had apparently normal enamel. It has been suggested that AMBN may function in bone development. No clinically obvious bone or other co-segregating health problems were identified in the family investigated. This study confirms for the first time that *AMBN* mutations cause non-syndromic human AI and that mouse models with disrupted *Ambn* function are valid.

## INTRODUCTION

Formation of dental enamel (amelogenesis) results in the most mineralized tissue in the human body. Enamel can last a lifetime even though it does not have the capacity for cellular repair. This longevity reflects that enamel predominantly consists of highly elongated crystals of substituted calcium hydroxyapatite that are arranged within prisms (rods), which each chart the pathway of a single ameloblast. This provides a tissue architecture that allows enamel to resist the mechanical stresses associated with daily masticatory function (1).

This developmental endpoint requires the deposition of an organic enamel matrix into a discrete extracellular compartment within the developing tooth by epithelially derived ameloblasts, which are part of the enamel organ (2). The secreted organic matrix is predominantly amelogenin but also includes other less abundant, but functionally critical proteins, including ameloblastin (AMBN). Matrix secretion is coincident with calcium hydroxyapatite nucleation and early crystal growth. As ameloblasts secrete the matrix, there is instantaneous precipitation of thin immature enamel crystals. Matrix secretion ceases once the pre-determined enamel volume is completed. At this point the matrix is approximately one-third mineral by weight and mechanically weak. During a period of maturation, the enamel matrix is degraded and removed allowing the initial crystals to grow in width and thickness until the tissue is almost fully occluded by mineral (1). On completion of enamel formation the ameloblasts form the reduced enamel epithelium on the tooth surface, which is lost at the time of tooth eruption (3).

Amelogenesis imperfecta (AI) (MIM 104530) is the term given to a heterogeneous group of disorders characterized by failure of normal amelogenesis, with a reported prevalence of between 1/700 and 1/14 000 (4,5). AI may have a profoundly negative functional and emotional impact on individuals that may include pain and difficulty in eating as well as social avoidance, distress and low self-esteem (6). Dental care can be challenging and protracted.

Based on clinical features, AI can be simply classified as hypoplastic or hypomineralized with the key distinction being the volume of enamel matrix secreted during amelogenesis. Hypoplastic AI is characterized by a grossly diminished enamel volume reflecting early failure of the enamel organ. In contrast, in hypomineralized AI there is near normal enamel volume that is characterized by failure of normal biomineralization. Within this subtype, hypomaturation AI and hypomineralized AI are characterized by brittle and soft enamel, respectively. Both lead to premature enamel loss following tooth eruption. To date, mutations in very few genes have been identified as a cause of autosomal dominant, autosomal recessive or X-linked AI in the absence of other co-segregating abnormalities (7–15).

AMBN (MIM *601259) is the second most abundant enamel matrix protein after amelogenin, which is coded by *AMELX* (MIM *300391) and *AMELY* (MIM *410000). Amelogenin accounts for up to 90% of the enamel matrix protein content. AMBN is known to play key roles in amelogenesis although its exact role remains to be fully elucidated. Poor quality enamel is characteristic of mouse models with deletion of *Ambn* exons 5 and 6 (*Ambn*^−5,6/−5,6^) (16,17) and Ambn over-expression (18). These models give insight into the underlying failure of the enamel organ and the associated AI. *AMBN* is therefore an excellent candidate AI gene, and human AI cohorts have been screened for *AMBN* mutations (19–25). Unlike *AMELX,* however, no disease causing genetic *AMBN* variants have so far been reported. Here we report for the first time that *AMBN* mutations cause non-syndromic human AI and confirm that mouse models with disrupted *Ambn* function are valid.

## RESULTS

### Patient phenotype and SNP mapping

**Figure 1.**
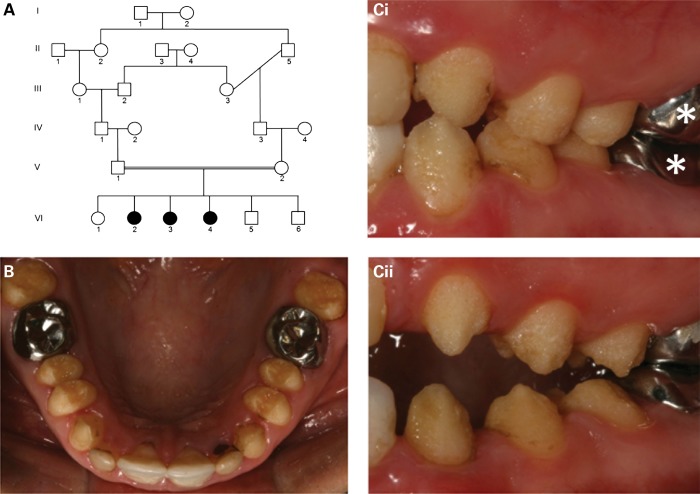
Family pedigree and dental phenotype. (**A**) Pedigree of the Costa Rican family investigated. Affected family members are shaded. (**B**) The permanent teeth of affected individuals (VI:3 presented) were characterized by generalized hypoplastic AI involving all teeth. (**C**) The enamel surface was rough, but hard (VI:3 and VI:4 presented in Ci and Cii, respectively). Note: some teeth have been restored with metal or tooth-coloured restorations (examples marked*).

We identified a consanguineous Costa Rican family with generalized hypoplastic AI involving the primary and secondary dentitions, in the absence of co-segregating health problems (Fig. 1). DNA was obtained and extracted from eight samples using Oragene DNA collection tubes (DNA Genotek, Kanata, Canada) according to the manufacturer's protocol.

Genomic DNA from individuals VI:2, VI:3 and VI:4 was analysed using Affymetrix 6.0 SNP microarrays. Two shared regions of homozygosity were identified using SNP Viewer (http://sourceforge.net/projects/snpviewer/), these being a 14.5 Mb region on chromosome 1p (Chr1: 11 698 902–26 171 473) and a 12.2 Mb region on chromosome 4q (Chr4: 71 230 491–83 403 217). While no known AI genes lay within the chromosome 1 region, the chromosome 4 region contains a number of genes encoding proteins with essential roles in tooth development, including two, *ENAM* and *C4orf26,* already implicated in AI. Sequencing of both genes however revealed no mutations.

### Whole-exome sequencing

We therefore performed whole-exome sequencing using a SureSelect All Exon XT reagent (Agilent Technologies, Edinburgh, UK) on DNA from individual VI:2. Following alignment, processing and duplicate removal, a mean depth of 40.12 reads per base was observed in the shared homozygous regions, with 95.5% of coding bases covered by at least 5 reads. Indel and single nucleotide variants within the candidate regions were called in the VCF format using the UnifiedGenotyper function of Genome Analysis Toolkit (GATK), identifying a total of 672 variants passing standard GATK filters. Using the dbSNP database at NCBI (http://www.ncbi.nlm.nih.gov/projects/SNP/), any variants present in dbSNP129 or with a minor allele frequency ≥1% in dbSNP137 were filtered out as polymorphisms, leaving just 46 variants. After filtering for nonsynonymous variants, exonic insertions or deletions or variants at splice consensus sites, only a single variant remained (chr1:17026040_17026041insGCGGCGGCGGCGGCA) causing an in-frame insertion of 15 bp in the *Espin Pseudogene* (*ESPNP*). However, analysis of the surrounding sequence revealed that the insertion was within a simple (CGG)*n* repeat with multiple reported repeat number polymorphisms in dbSNP. The *ESPNP* variant was thus considered an unlikely cause of disease in this family.

To confirm that sequence reads for all exons in the homozygous regions were present, the ‘DepthOfCoverage’ function of GATK was used to analyse the average read-depth across each exon. Using an in-house perl script, the results were compared with five unrelated exomes processed in an identical manner as the sample from individual VI:2. This revealed a lack of reads (average 0.56 reads/base) in exon 6 of the *AMBN* gene (NM_016519), compared with an average of 230 reads/base (range 200–269) in the five comparison exomes. Investigation of the remaining exons of *AMBN* revealed that read depth for these exons in individual VI:2 was similar to that in comparison exomes.

### PCR and sanger sequencing

**Figure 2.**
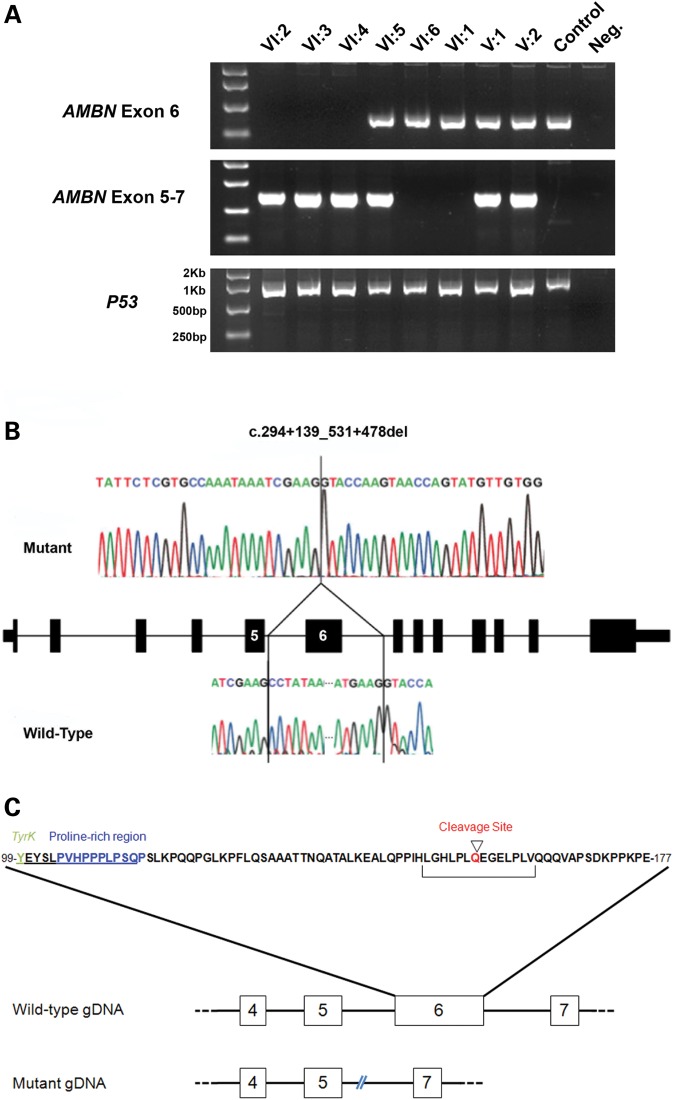
Genotyping of the mutation and identification of the deletion breakpoints. (**A**) PCR analysis of *AMBN* exon 6 in members of the family investigated. Amplification of exon 6 alone was only observed in individuals with clinically normal teeth and a normal *AMBN* genotype, whereas PCR with primers spanning from exons 5 to 7 amplified a product of 794 bp in affected and carrier individuals only, reflecting loss of exon 6. Bands obtained with primers amplifying P53 are shown as a positive control for each sample. (**B**) Sequence electropherograms of mutant and wild-type alleles to identify the deletion breakpoints. Comparison of the mutant sequence with a control sequence revealed a deletion of 2347 bp spanning exon 6 and the majority of the surrounding introns either side. The deletion begins 114 bp from the end of exon 5. (**C**) Wild-type amino acid sequence of AMBN exon 6. Important domains, which are likely to play a key role in the function of the mature protein, are labelled. Schematics of the exon-intron layout of wild-type and the mutant genomic DNA sequences are also shown, showing the lack of exon 6 and the approximate break-points with respect to the local exons.

To investigate the lack of reads further, PCR amplification using primers designed to amplify exon 6, plus 50 bp of flanking intron, was performed. The results corroborated the exome data in that no product was observed in the affected members of the family, whereas amplification was successful in unaffected family members. A control PCR, designed to amplify part of the *P53* gene, was successful in all affected and unaffected samples (Fig. 2A). To determine the cause of this result, a long range PCR was undertaken with primers designed to span from exons 5 to 7 in *AMBN* genomic DNA. Sequencing of the resulting product revealed a deletion of 2347 bp (c.294 + 139_531 + 478del) encompassing all 237 bp of exon 6 and 2110 bp of flanking sequence either side (Fig. 2B). To confirm the loss of exon 6, and to determine the effect this has on transcription of *AMBN* mRNA, patient leukocyte cDNA was obtained. While cDNA amplification with control primers was successful, amplification of *AMBN* products failed in both patient and control cDNA, most probably due to insufficient expression of the mRNA in blood.

The deletion identified is predicted to create an in-frame deletion of 79 amino acids (p.Tyr99_Glu177del), shortening the protein from 447 to 368 amino acids (Fig. 2C). Based on this result, we screened all coding exons and flanking introns of *AMBN* in an additional 13 recessive hypoplastic AI samples, but no further mutations were identified.

### Enamel phenotyping

On clinical examination of the teeth, the three family members homozygous for the mutation (VI:2, VI:3 and VI:4) exhibited rough hypoplastic AI of the permanent dentition. The child heterozygous for the mutation (VI:5) exhibited some minor enamel defects that were very different from the hypoplastic AI of the three affected siblings. Individuals VI:1 and VI:6 did not carry the mutation and exhibited a normal dentition (Supplementary Material, Fig. S1).

**Figure 3.**
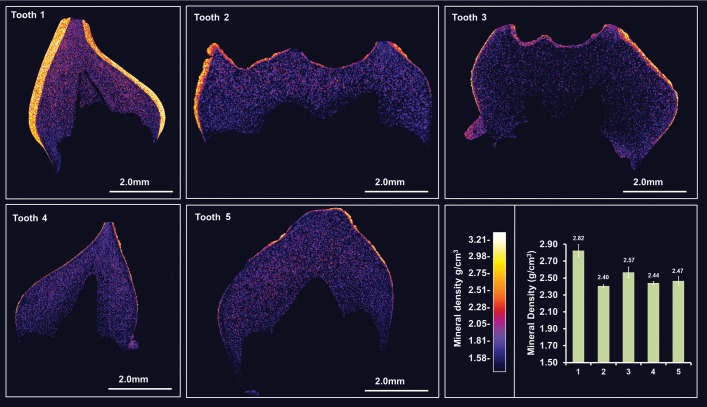
High resolution X-ray CT analysis of exfoliated teeth **1–5**. Typical CT sections through the teeth are presented using false colour calibrated with respect to mineral density to generate mineral density maps. Mean enamel mineral density for each tooth is also shown graphically. Tooth 1 exhibits an enamel layer apparently normal in structure and density. Teeth 2 and 3 exhibit an obvious enamel covering though it is thinner in many areas, chipped, absent in places and is significantly reduced in mineral density compared with tooth 1 (*P* < 0.0001). Teeth 4 and 5 exhibit an even thinner covering of enamel with enamel absent in some areas. Again enamel mineral density is significantly lower than in tooth 1 (*P* < 0.0001). The only non-significant differences in enamel mineral density occur between teeth 4 and 5. Videos of 3D rendered CT data showing surface detail and internal structure of all teeth are available as Supplementary Material.

Five deciduous teeth (teeth 1–5) retained by the family after natural exfoliation were made available for phenotypic characterization. No photographs of the deciduous teeth when present in the mouths were available for review. The teeth could therefore not be matched to individual family members VI:1–VI:6 and accordingly, phenotypes could not be matched unambiguously with a specific genotype. High resolution-CT scanning (Fig. 3 and Supplementary Material, Videos 1–5) identified that tooth 1 exhibited a normal enamel layer in terms of thickness and mineral density [density = 2.82 g ± 0.073 g/cm^3^ compared with 2.69–2.92 g/cm^3^—the range previously reported for deciduous enamel (26)] consistent with originating from either individual V:I or VI:6 (both normal genotype). The enamel covering teeth 2 and 3 was pitted and varied in thickness over the crown from near normal to areas where thickness was reduced to tens of microns and in some places was absent altogether. The enamel covering teeth 4 and 5 was thinner (∼50 µm or less) or absent to a greater degree than in teeth 2 and 3. Mean mineral density was significantly reduced in teeth 2–5 relative to tooth 1 (Fig. 3).

**Figure 4.**
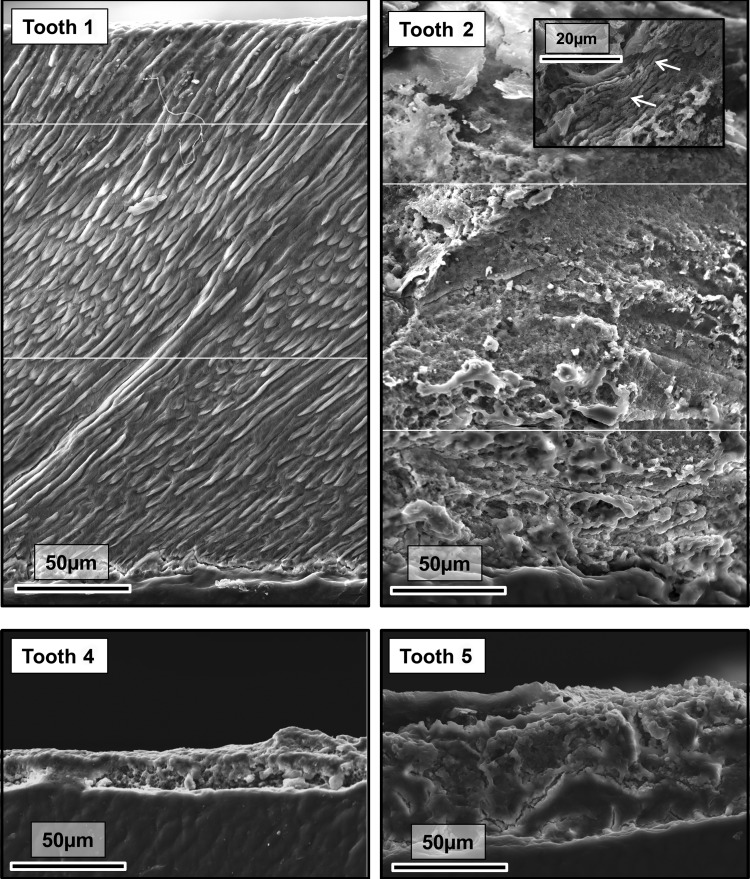
SEM of representative exfoliated teeth. Tooth **1** exhibits normal enamel architecture comprising prisms (rods) of individual enamel crystallites classically described in numerous research publications and text books. The SEM representative of the thicker enamel found covering tooth **2** is lacking the obvious long range order and structure that characterizes normal enamel. However, very occasional areas exhibiting prism-like structures are present (indicated by arrows on inset micrograph). Where present, the enamel covering teeth **4** and **5** is thin and exhibits no structural similarities to normal enamel. The white horizontal lines show where separate micrographs have been collaged to create a wider field of view.

On SEM, tooth 1 exhibited the prismatic structure characteristic of normal human enamel (Fig. 4). Thicker areas of enamel on the abnormal teeth (e.g. tooth 2) lacked normal prismatic structure. However, occasionally structures were visible that resembled poorly formed prisms (inset; Fig. 4 tooth 2). Reduced enamel thickness and absence of any normal architecture was even more pronounced in enamel covering teeth 4 and 5. Differential post eruptive wear may have affected the enamel thickness as measured, but would not account for lack of the normal prismatic enamel structure in teeth 2–5. No permanent teeth were available for analysis and so it is unknown what impact the *AMBN* deletion (heterozygous or homozygous) would have on the ultrastructure of the enamel recorded in the clinical photographs presented.

## DISCUSSION

We have identified a family from Costa Rica segregating hypoplastic AI in an autosomal recessive manner. SNP genotyping revealed a region on chromosome 4 shared by all affected individuals and whole-exome sequencing showed a lack of reads across AMBN exon 6. Further investigation showed this to be due to a 2347 bp deletion encompassing the whole of exon 6 and flanking intronic sequence either side. A screen of 13 additional hypoplastic AI families revealed no further mutations.

A mouse model homozygous for *Ambn* lacking exons 5 and 6 (*Ambn*^−5,6/−5,6^) has a similar phenotype to the most severe phenotype reported here with secretion of only a thin dysplastic layer of mineralized tissue that is rough and pitted and devoid of normal prismatic structure (16,17). Heterozygous *Ambn*^+5,6/−5,6^ mice had prismatic enamel of near normal thickness but that was significantly less-well mineralized compared with wild-type (16,17). Likewise here, mineral density is significantly decreased in all teeth 2–5 compared with unaffected tooth 1. Heterozygous *Ambn*^+5,6/−5,6^ mice had an ordered prismatic structure. In contrast, the severely disrupted prismatic structure in teeth 2 and 3 led us to tentatively conclude that these may be from family member VI:5 who is heterozygous for the *AMBN* mutation.

AI may co-segregate with other clinical phenotypes that may not always be obvious. No co-segregating conditions were identified from speaking with the family. Potential roles for AMBN in bone development and remodelling /repair have been reported (27,28). However, the role of AMBN in bone is unconfirmed and mouse models of loss of *Ambn* function did not have obvious bone defects including abnormalities of bone remodelling or repair (16,17,29,30). This is in keeping with the findings reported here. On discussion with the family there were no obvious bone abnormalities and nothing was identified that warranted further, detailed clinical evaluation. The oldest affected individual was entering adulthood when assessed and any effect of the *ABMN* mutation may have on bone may not become apparent until later in life.

The precise mechanisms underlying the enamel defect in this family are unclear. Although the deletion is in-frame, the loss of 79 amino acids in a part of AMBN believed to have important functions can be expected to have a significantly detrimental impact on AMBN function.

In wild-type rodents expression of Ambn by ameloblasts is highest during the secretory stage of amelogenesis and diminishes during the maturation phase (28,31). Following secretion, Ambn is cleaved into C- and N-terminus fragments by enamel matrix proteases including Mmp20. The basic 17KDa N-terminus fragment and its processing products are highly aggregative and can be immunolocalized to the sheath space surrounding enamel prisms. The acidic C-terminus is degraded and removed from the matrix soon after secretion. It has been suggested that the N-terminal fragments have a key role in the formation of the prism sheath (32,33). Biological activity has also been associated with 17 KDa N terminal pig AMBN with native and synthetic 17 KDa AMBN peptides being able to induce alkaline phosphatase activity in human periodontal ligament cells; this activity appears to reside in a domain (-VEGPMVQQQVAPSEK-) near the C-terminal of the molecule (34). The corresponding region in human AMBN is deleted in the human *AMBN*^−6/−6^ mutation reported here and in the Ambn^−5,6/−5,6^ mouse mutant previously reported. It is unknown if the N-terminal 17 kDa fragment has any role in inducing alkaline phosphatase expression during amelogenesis but the enamel organ expresses tissue non-specific alkaline phosphatase (35) and alkaline phosphatase activity is readily detected in developing enamel matrix (36).

Msx2 is a transcriptional repressor of mouse *Amelx* (37). In Ambn^−5,6/−5,6^ mice ameloblast Msx2 expression remains abnormally elevated during the secretory stage of amelogenesis and appears to dramatically down-regulate Amelx expression without affecting expression of other enamel matrix proteins (30). However, since Amelx accounts for up to 90% of the enamel matrix protein its down-regulation due to elevated Msx2 activity might partially account for the thin enamel in Ambn^−5,6/−5,6^ mice and possibly AMBN^−6/−6^ teeth 4 and 5 reported here.

AMBN has also been implicated in cell adhesion. In Ambn^−5,6/−5,6^ mice, the ameloblast monolayer detaches from the enamel matrix (30) and the specialized ameloblast process (Tomes process) responsible for orchestrating prismatic enamel formation fails to develop (16). The ameloblasts lose their normal morphology and begin proliferating abnormally to produce a multi-cell layer (30) and normal enamel matrix secretion fails resulting in the hypoplastic AI phenotype. Similar phenotypes are also observed in animal models over-expressing *Ambn* (18). The mechanisms responsible for this detachment have yet to be elucidated. AMBN has been reported to interact with cells via binding sites for fibronectin (38) and heparin (39) but the role played by such interactions in amelogenesis is unclear.

In summary, the identification of a homozygous 2347 bp genomic deletion in *AMBN* identifies for the first time that *AMBN* mutations cause autosomal recessive hypoplastic human AI. The mechanisms causing AI in this case are unclear but may involve the critical role of AMBN in ameloblast attachment, maintenance of the ameloblast phenotype and repression of transcription factors that otherwise down-regulate amelogenin expression. Although detailed analysis of skeletal tissues was not undertaken, there was an absence of obvious bone or other defects associated with this mutation in these young adults. A heterozygous carrier for the 2347 bp genomic deletion in *AMBN* was identified. Although there were some minor enamel abnormalities in the permanent dentition, this individual did not have hypoplastic AI. Comparison of the enamel phenotype of exfoliated deciduous teeth suggests that mouse models with disrupted *Ambn* function are valid.

## MATERIALS AND METHODS

### Patients

Affected individuals and family members were recruited following informed consent in accordance with the principles outlined by the declaration of Helsinki, with local ethical approval. Genomic DNA samples were obtained using Oragene^®^ DNA sample collection kits (DNA Genotek, ONT, Canada) according to the manufacturer's instructions.

### SNP analysis

Genomic DNA from three affected individuals were genotyped using Affymetrix 6.0 SNP microarrays by AROS Applied Biotechnology (Aarhus, Denmark). Shared regions of homozygosity were identified using SNP Viewer (http://sourceforge.net/projects/snpviewer/).

### Whole-exome sequencing and analysis

DNA from individual VI:2. Three micrograms of genomic DNA were processed according to the Agilent SureSelect Library Prep protocol (Agilent Technologies, CA, USA). Sequencing was performed using a 100 bp paired-end protocol on an Illumina HiSeq 2500 sequencer (Illumina, CA, USA). The resulting fastq files were aligned to the human reference genome (GRCh37) using the Bowtie2 software (40). This alignment was processed in the SAM/BAM format using Picard and The GATK java programs (41–43).

### Tooth ultrastructure analysis

Five deciduous teeth (teeth 1–5) retained by the family after natural exfoliation were made available for phenotypic characterization. No photographs of the deciduous teeth when present in the mouths were available for review. These teeth were subjected to high resolution CT using a Skyscan 1172 (Brucker, UK) operated at 100 kV; source current of 100 µA, using a Al/Cu filter to reduce beam hardening. CT slices were reconstructed using the Skyscan Recon software and rendered as 3D videos using the Skyscan CTVox software. The CT images were calibrated using a calibration phantom comprising of a section of a mouse incisor and the mean mineral density of enamel was determined using the ImageJ software (http://imagej.nih.gov/ij/) by measuring the mineral density of any surface material >2 g/cm^3^ present on 10 evenly spaced CT slices taken through each tooth. Calibrated colour contour maps of mineral density were also generated using ImageJ.

For SEM, teeth were sectioned with a diamond disk and the cut surfaces smoothed using fine carborundum paper. Specimens were etched in 30% phosphoric acid for 20 s followed by thorough rinsing in excess distilled water. Teeth were dried overnight under vacuum and sputter coated with gold. Specimens were observed using a Hitachi S-3400N scanning electron microscope operated at an accelerating voltage of 20 kV.

## SUPPLEMENTARY MATERIAL

Supplementary Material is available at *HMG* Online.

## FUNDING

This work was supported by the Wellcome Trust (grant number 093113). D.A.P. is funded by a Sir Jules Thorn Award for Biomedical Research (grant number JTA/09). J.K. is supported by the NIJR Leeds Musculoskeletal Biomedical Research Unit. Funding to pay the Open Access publication charges for this article was provided by the Wellcome Trust.

## Supplementary Material

Supplementary Data
