# Supplementary material for: Deletion of ameloblastin exon 6 is associated with amelogenesis imperfecta
Source: Hum Mol Genet. 2014 May 23;23(20):5317–24. doi: 10.1093/hmg/ddu247 (PMC4168819; doi:10.1093/hmg/ddu247)
Supplement: Supplementary Data [file supp_23_20_5317__index.html]

Deletion of ameloblastin exon 6 is associated with amelogenesis imperfecta — Supplementary Data 

# Deletion of ameloblastin exon 6 is associated with amelogenesis imperfecta

## Supplementary Data

Supplementary Data

**Files in this Data Supplement:**

- Supplementary Data - Doc file
- Supplementary data - docx file
- Supplementary Video 1 - avi file
- Supplementary Video 2 - avi file
- Supplementary Video 3 - avi file
- Supplementary Video 4 - avi file
- Supplementary Video 5 - avi file
