# Supplementary material for: Deletion of ameloblastin exon 6 is associated with amelogenesis imperfecta
Source: Hum Mol Genet. 2014 May 23;23(20):5317–24. doi: 10.1093/hmg/ddu247 (PMC4168819; doi:10.1093/hmg/ddu247)
Supplement: Supplementary Data [file supp_ddu247_ddu247supp.doc]

**Supplementary Data**

**Homozygous loss of Ameloblastin exon 6 results in isolated hypoplastic Amelogenesis Imperfecta**

James A. Poulter, Gina Murillo, Steven J. Brookes, Claire E. L. Smith, David A. Parry, Sandra Silva, Jennifer Kirkham, Chris F. Inglehearn and Alan J. Mighell.

**Supplementary Table 1. Oligonucleotide primers used for sequence analysis of AMBN.**

| **Primer** | **Forward (5’-3’)** | **Reverse (5’-3’)** |
| --- | --- | --- |
| Exon 1 | TCTTCCCTGAATGAGAAGTACAGA | TTCAGCTACTGGTGACAAATGAA |
| Exon 2 | GAGCAGAGACTCAGGCTCATTT | TCCCTTCAGTGTTGTTTGGA |
| Exon 3 | GCAATATCCTACGCCCAATG | GGAAAACTGAGAAGCACACGA |
| Exon 4 | CAAACTTTGTGTTGATAATGTCAAAT | TGATGAATTATAATGCTGCTGCTA |
| Exon 5 | CAACATCACTTTTGCTTTTGC | TCCAGGATGTAGCCTGCTTT |
| Exon 6 | CCAAGCCCCTTTGTTTAGAA | CCCTGCAAATTCGTTTTCTT |
| Exon 7-9 | CCTCCCAAAACACTAAAAGAAA | TCATGGATAAATGGGACAATGA |
| Exon 10-11 | TGGGTTCCTTTGTTCTCTTAAA | TGTTCAGAGAATGTTTATTTGGAATC |
| Exon 12 | TCACCTCTCCACTTTGCCTC | AGACGTCTCTTGCAGCCAGT |
| Exon 13a | GACCAGGTATAGCTGCATGG | CTGGGGAGTCCCTTCATCTT |
| Exon 13b | TTACAGAGCTAGAACCTGCTCC | GCTGCAGAAGGGTAATAAGTGTTT |

**Supplementary Table 2. Oligonucleotide primers used for analysis of AMBN deletion.**

| **Primer** | **Forward (5’-3’)** | **Reverse (5’-3’)** |
| --- | --- | --- |
| Exons 5-7 | TCCCACCACATTCCTCTCTT | GATGGACCTTGTGGATCAGC |
| P53 control | AGATATTCCCCTGCCCTCAACA | CTGGAGTCTTCCAGTGTGAT |

**Supplementary Figure 1 - Dental phenotype of other siblings of the affected individuals.** (A) The teeth of individual IV:5, who is a carrier for the *AMBN* deletion, exhibit near normal morphology, but with some minor enamel defects. (B) & (C) The teeth of individuals IV:6 (B) and IV:1 (C) have normal clinical appearances reflecting that they have a wild type *ABMN* genotype. Note: The mild white enamel flecking evident on the teeth of the IV:1, IV:5 and IV:6 are not thought to be related to the main finding of this study.
