# Supplementary material for: Deletion of ameloblastin exon 6 is associated with amelogenesis imperfecta
Source: Hum Mol Genet. 2014 May 23;23(20):5317–24. doi: 10.1093/hmg/ddu247 (PMC4168819; doi:10.1093/hmg/ddu247)
Supplement: Supplementary Data [file supp_ddu247_ddu247supp_data.docx]

**Deletion of ameloblastin exon 6 is associated with amelogenesis imperfecta**

James A. Poulter, Gina Murillo, Steven J. Brookes, Claire E. L. Smith, David A. Parry, Sandra Silva, Jennifer Kirkham, Chris F. Inglehearn and Alan J. Mighell.

**Supplemental videos A-E**

Video files of 3D rendered CT scans of teeth A-E showing CT sectioning through the teeth. Note the teeth are not necessarily to scale. False colour is used to highlight the enamel and dentine components of the tooth but is not intended to provide quantitative information on mineral density.
